# Immune Cytolytic Activity Is Associated With Genetic and Clinical Properties of Glioma

**DOI:** 10.3389/fimmu.2019.01756

**Published:** 2019-08-02

**Authors:** Zhi-liang Wang, Zheng Wang, Guan-zhang Li, Qiang-wei Wang, Zhao-shi Bao, Chuan-bao Zhang, Tao Jiang

**Affiliations:** ^1^Department of Molecular Pathology, Beijing Neurosurgical Institute, Capital Medical University, Beijing, China; ^2^Department of Neurosurgery, Beijing Tiantan Hospital, Capital Medical University, Beijing, China; ^3^China National Clinical Research Center for Neurological Diseases, Beijing, China; ^4^Center of Brain Tumor, Beijing Institute for Brain Disorders, Beijing, China

**Keywords:** glioma, immune cytolytic activity, genetic, clinical, survival

## Abstract

**Background:** Immunotherapy provided unprecedented advances in the treatment of several previously untreated cancers. However, these immunomodulatory maneuvers showed limited response to patients with glioma in clinical trials. Our aim was to depict the immune characteristics of glioma with immune cytolytic activity at genetic and transcriptome levels.

**Methods:** In total, 325 gliomas from CGGA dataset as training cohort and 699 gliomas from TCGA dataset as validation cohort were enrolled in our analysis. We calculated the immune cytolytic activity for 1,000 of gliomas. The characteristics of immune cytolytic activity in gliomas were interpreted by the corresponding clinical, molecular genetics and radiological information.

**Results:** We found that immune cytolytic activity was highly associated with molecular, clinical, and edema extent. High cytolytic activity gliomas were more likely to be diagnosed as glioblastoma and might be a potential marker of mesenchymal subtype. Moreover, those gliomas exhibited significantly increased copy number alterations including recurrent focal amplifications of PDGFA and EGFR, as well as recurrent deletions of CDKN2A/B. Subsequent biological function analysis revealed that the immune response and immune checkpoints expression were significantly correlated with the cytolytic activity of gliomas. Immune cytolytic activity was significantly positively associated with the extent of peri-tumor edema and was independently correlated with reduced survival time.

**Conclusion:** Our results highlighted the immunoregulatory mechanism heterogeneity of gliomas. Cytolytic activity, indirectly reflected by the extent of peri-tumor edema, may provide a potential index to evaluate the status of immune microenvironment and immune checkpoints in glioma, which should be fully valued for precision classification and immunotherapy.

## Introduction

Diffuse gliomas, accounting for the majority of adult malignant brain tumors (1), are mainly divided into five strategies defined by both histological and molecular pathological features (2). The standard treatment for glioma consists of maximal surgical resection, radiotherapy, and concomitant and adjuvant chemotherapy with temozolomide (3, 4). Despite considerable advances in the understanding of the genetic and clinical characteristics of gliomas, contemporary methods and techniques are not curative for the durable remissions of glioma patients. The molecular subtypes of glioma, based on the integrated multi-omics analysis, may bring us opportunities to achieve high-precision targeted therapy for individual patients.

The immune system is a crucial regulator to facilitate or inhibit tumor biological functions (5, 6). Recent immunotherapy drugs, such as targeting the immune checkpoint pathways PD1/PD-L1, CTLA4, and LAG3, are exhibiting the significant effectiveness on a broad of cancers (7). However, those immunotherapy drugs showed limited efficacy in treating majority of patients with glioma in many clinical trials (8). The microenvironment of glioma is characterized by the production of immunosuppressive cytokines, inhibition of T-cell proliferation and effector responses and tissue hypoxia. Provocative evidence suggests that the extent of cytotoxic T cell could significantly affect overall survival (OS) of glioma patients (9). At present, the determinants of immune response and immune checkpoint activity in glioma are poorly understood.

A simple and quantitative formula to estimate the immune cytolytic activity (CYT) of glioma was proposed by Rooney et al. (10). The CYT is assessed by granzyme A (GZMA) and perforin-1 (PRF1), which is dramatically reflected the activation of CD8+ T cell and immune status (11, 12). The research about molecular and genetic properties of CYT on glioma was rarely studied. Therefore, in this article, we explored the RNA sequence data from 1,024 glioma specimens to explore the genetic and clinical properties of CYT in glioma. We found that CYT was an unfavorable prognostic biomarker for patients with glioma. We also demonstrated that glioma peri-tumor edema showed a significantly positively relationship with CYT. These results may give us the opportunity to simply detected the immune characteristics non-invasively and may help guide the development of effective immune therapy in glioma.

## Methods

### Samples and Datasets

This study retrospectively enrolled RNA sequencing data of 325 glioma patients, ranging from WHO grade II–IV, and corresponding clinical information from the Chinese Glioma Genome Atlas (CGGA) database (www.cgga.org.cn) as training cohort. The RNA sequencing data from the Cancer Genome Atlas (TCGA, https://portal.gdc.cancer.gov/) of 699 glioma patients, ranging from grade II–IV, and corresponding clinical information were collected as validation cohort. Moreover, we collected another 20 patients for independent clinical validation. The OS was calculated from the data of initial diagnosis until death or the last follow-up. Thus, in total 1,024 glioma patients were evaluated in this study. Information on these patients are available from corresponding data portal.

### Isocitrate Dehydrogenase (IDH1/2) Mutations Detection

IDH mutations typically leading to the replacement of an arginine with a histidine at amino acid residue 132 of the protein (R132H). The mutation results in a loss of normal enzymatic function and the abnormal production of 2-hydroxyglutarate (2-HG) (13). In CGGA cohort, IDH1/2 mutations were detected by DNA pyro-sequencing as previous reported (14). And the IDH1/2 mutations information were downloaded from TCGA website in TCGA cohort.

### Cytolytic Activity and Biological Analysis

We obtained Fragments Per Kilobase Million (FPKM) values of glioma samples from two datasets. The FPKM values was transformed into Transcript Per Million (TPM) values with following formula:

$$TPM=\frac{FPKM_{i}}{\sum_{i=1}^{n}FPKM_{i}}$$

The CYT was calculated as the geometric mean of the GZMA and PRF1 expression in TPM (10). Significance analysis of microarrays (SAM) was applied to identify the differentially expressed genes based on the threshold of fold change more than 2 and false discovery rate (FDR) value < 0.01 adjusted by 1,000 times permutation test. Gene ontology (GO) analysis was performed in DAVID (http://david.abcc.ncifcrf.gov/home.jsp) for functional annotation of the genes.

### Identification of Peri-Tumoral Edema

MR images of patients from the CGGA and TCGA databases were obtained from the CGGA imaging database (http://www.cgga.org.cn) and the Cancer Imaging Archive (http://www.cancerimagingarchive.net), respectively. Tumor contrast enhancement was assessed by two experienced neuroradiologists blinded to the patients' clinical information. A third senior neuroradiologist re-examined the images and determined which should be used if the types of enhancement identified by the first two neuroradiologists were inconsistent. Briefly, a small (or no) region of edema (-) was defined as edema extending ≤1 cm from the margin of the tumor based on T2-weighted images; otherwise edema was graded as moderate to severe (+) (15).

### Statistical Analysis

Difference in variables between groups were tested by the Student's *t*-test or chi-squared test, as appropriated. The Kaplan-Meier curves were employed to estimate overall survival distribution. Log-rank test was applied to compare the statistic difference of survival curves between two groups. All figures and statistical analysis were conducted using R software (version 3.4.2; http://www.R-project.org) and GraphPad Prism 7.0 software. A *p* < 0.05 was considered as statistically significant. All statistical tests were two-sided.

## Results

### Association of CYT With Clinical and Molecular Characteristic

**Figure 1:**
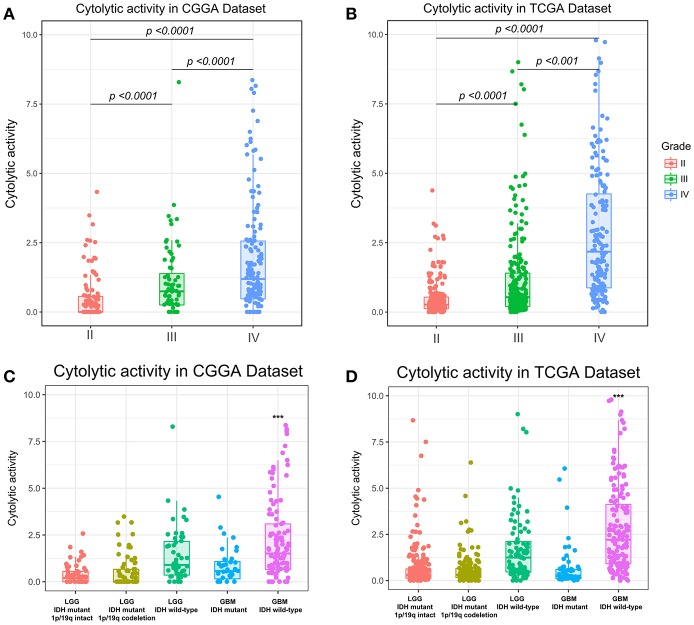
Immune cytolytic activity was significantly increased in WHO IV and GBM-IDH wild type gliomas in CGGA **(A,C)** and TCGA **(B,D)**. *p < 0.05, **p < 0.01, ***p < 0.001, and ****p < 0.0001, respectively.

To assess the stability and quality of CYT, we profiled the distribution of GZMA and PRF1 using their RNA expression data in CGGA and TCGA datasets, respectively. As shown in Figure S1, GZMA and PRF1 exhibited significantly positively correlation relationship in CGGA (*r* = 0.57, *p* < 0.0001) and TCGA (*r* = 0.7, *p* < 0.0001) datasets. Due to the intratumor heterogeneity and plasticity of glioma, we investigated the relationship of CYT and previous widely accepted prognostic and predictive factors, including grade, IDH status, and 1p/19q status. In CGGA dataset, cytolytic activity was significantly increased with increasing grade and the CYT exhibited the highest value in glioblastoma (Figure 1A). The similar result was well validated in TCGA dataset (Figure 1B). Moreover, IDH wildtype glioblastoma showed the highest CYT values (*p* < 0.001) when comparing to other four subtypes of glioma in both CGGA (Figure 1C) and TCGA (Figure 1D) datasets.

In addition, we also explored that CYT was highly enriched in IDH wild-type glioma group in both CGGA (Figure S2A, *p* < 0.0001) and TCGA datasets (Figure S2B, *p* < 0.0001). Meanwhile, we found CYT value was significantly upregulated in 1p/19q intact glioma group than 1p/19q co-deleted group in both CGGA (Figure S2C, *p* < 0.0001) and TCGA datasets (Figure S2D, *p* < 0.0001). These findings indicated that cytolytic T-cell-related immune activities were more prevalent in these relatively malignant subtypes of glioma.

### CYT Was a Potential Marker for Mesenchymal Subtype

**Figure 2:**
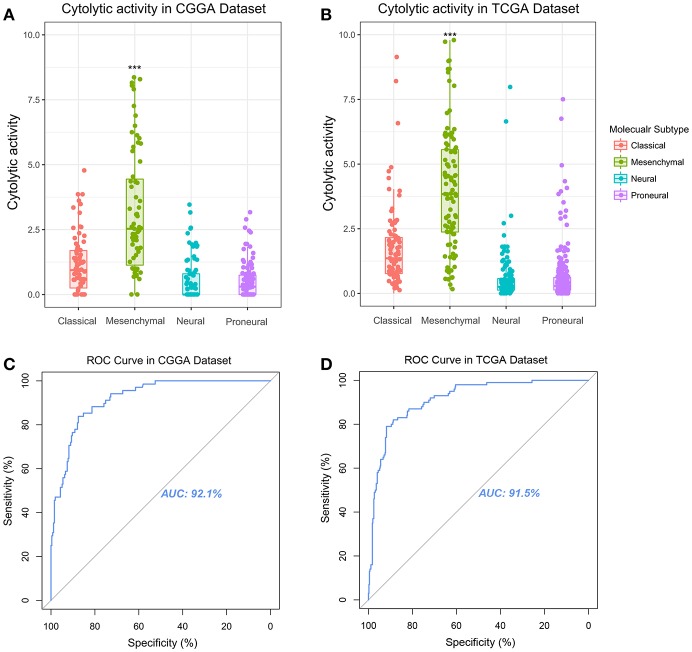
Immune cytolytic activity was significantly upregulated in the mesenchymal subtype of glioma in CGGA **(A)** and TCGA **(B)**. Receiver operating characteristic (ROC) curve for mesenchymal subtype prediction in CGGA and TCGA datasets. ROC curve analysis showed that CYT had highly sensitivity and specificity to predict mesenchymal subtype in CGGA and TCGA database. Area under curve (AUC) was 0.921 **(C)** and 0.915 **(D)**, respectively. *P < 0.05, **P < 0.01, ***P < 0.001, and ****P < 0.0001, respectively.

Glioma can be classified into four molecular subtypes: classical, proneural, neural, and mesenchymal (16). The glioma patients in mesenchymal subtype group always confer aggressiveness, treatment resistance and worse prognosis (17). The core genes to identify the mesenchymal subtype glioma was shown in Table S1. To investigate the relationship of glioma molecular subtype and cytolytic T-cell activity, we compared the difference of CYT value between four molecular subgroups. In CGGA cohort, CYT values were significantly increased (*p* < 0.0001) in mesenchymal subgroup (Figure 2A). Meanwhile, this trend of CYT distribution was validated in TCGA cohort significantly (*p* < 0.0001) (Figure 2B). Then Receiver Operating Characteristic (ROC) curves were used to estimate the diagnostic ability of CYT to discriminate the true status of mesenchymal subtype. The area under the curve (AUC) was 92.1 and 91.5% in CGGA (Figure 2C) and TCGA (Figure 2D), respectively. Those results emphasized that CYT was closely connected with malignant progression of glioma and could serve as a biomarker for mesenchymal subtype.

### CYT Associated With Different Patterns of Genomic Alterations

**Figure 3:**
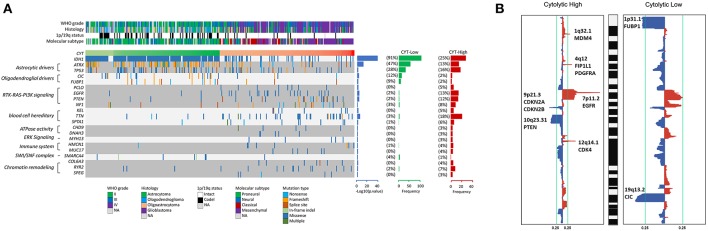
High cytolytic index is associated with increased copy number alterations in glioma. **(A)** Recurrently mutated genes. Mutation plot showing significantly mutated genes (SMGs, FDR < 0.05) in cytolytic subsets in the TCGA dataset. Recurrently mutated genes, which are grouped on the basis of molecular pathways, are shown on the left. Their mutation frequencies in CYT-low group tumors and in CYT-high group tumors were shown on the right. Each tumor's clinicopathological characteristics were shown at the top. **(B)** Significant copy number gains (red) and losses (blue) detected by analysis of the copy number data from TCGA cohorts using GISTIC 2.0. Candidate gene targets within the corresponding loci are also indicated.

To reveal the molecular mechanism underlying the cytolytic T-cell activity of glioma, we divided gliomas of TCGA dataset into CYT-high cohort (tumors in the top 25th percentile) and CYT-low cohort (tumors in the bottom 25th percentile). We identified the diversity mutational profiles characteristic of gliomas (Figure 3A). Overall, considering all 326 glioma samples, we confirmed 21 genes (Chi-square test, *p* < 0.05) significantly mutated in CYT-high or CYT-low cohorts. Generally, 91% glioma samples harbored IDH1 mutation, which mostly concurrent with either TP53 and ATRX mutations in astrocytoma samples or with CIC and FUBP1 mutations in oligodendroglioma samples in CYT-low cohort. Meanwhile, 16 genes were founded with significantly higher frequencies of nonsense, splice-site and frameshift mutations (*p* < 0.05) in CYT-high cohort, which could affect RTK-RAS-PI3K pathway, blood cell hereditary, ATPase activity, ERK pathway, immune system, SWI/SNF complex, and chromatin remodeling.

Besides coding mutations, we also estimated the copy number alterations information of gliomas between CYT-high and CYT-low cohorts (Figure 3B). The most commonly recurrent deletion events on chromosome 1p (ARID1A, CDKN2C, FUBP1) and chromosome 19q (CIC) were observed in gliomas of CYT-low cohort. Meanwhile, the recurrent focal amplifications of chromosome 1q32.1 (MDM4), 4q12 (FIP1L1, PDGFRA), 7p11.2 (EGFR), and 12q14.1(CDK4), and deletion regions of 9p21.3 (CDKN2A, CDKN2B) and 10q23.31(PTEN) were the most frequently identified in CYT-high cohort. In general, glioma samples in CYT-low cohort possessed fewer somatic copy number alterations (SCNA events) and copy number alterations than CYT-high cohort.

### CYT-Related Biological Processes

**Figure 4:**
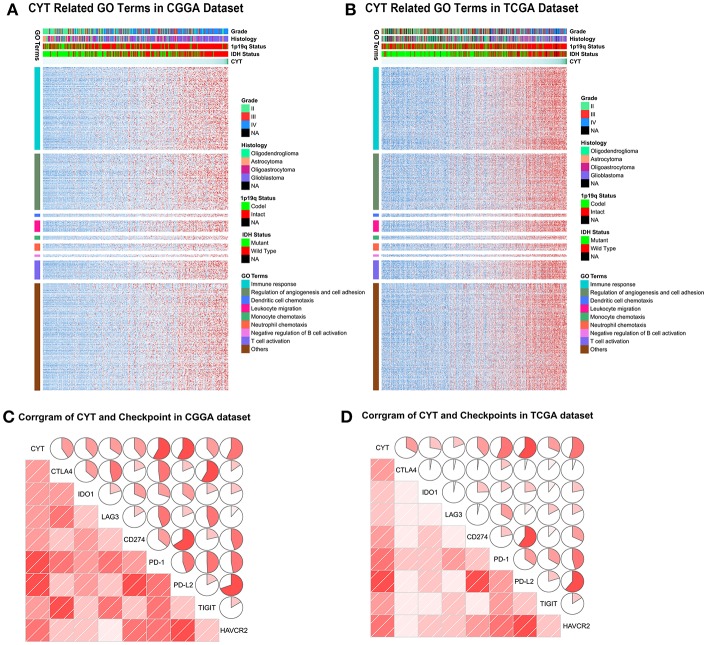
The biology function of immune cytolytic activity positively related genes in CGGA **(A)** and TCGA **(B)** cohorts. The biological functions related with immune response, regulation of angiogenesis and cell adhesion, dendritic cell chemotaxis, leukocyte migration, monocyte chemotaxis, neutrophil chemotaxis, T cell activation, and negative regulation of B cell activation. **(C,D)** CYT was significantly positively associated with immune checkpoints, including CTLA4, IDO1, LAG3, CD274, PD-1, PD-L2, TIGIT, and HAVCR2.

To depict the biological features correlated with cytolytic activity, we compared the gene expression profiles of glioma samples between CYT-low group and CYT-high group. Differentially expressed genes were selected with conditions of | log2 (fold-change) | >1 and adjusted *p* < 0.001. We found 354 down-regulated genes and 1,166 up-regulated genes strongly correlated with CYT in CGGA dataset (Figure S3A), while 534 down-regulated genes and 2,034 up-regulated genes strongly correlated with in TCGA dataset (Figure S3B). To yield an accuracy analysis, the differentially expressed genes which were shared by two datasets (847 up-regulated genes and 188 down regulated genes) were selected for Gene Ontology analysis with online methods (DAVID, https://david.ncifcrf.gov/). We found that genes positively correlated with cytolytic T-cell activity were more involved in immune response, regulation of angiogenesis and cell adhesion, dendritic cell chemotaxis, leukocyte migration, monocyte chemotaxis, neutrophil chemotaxis, T cell activation, and negative regulation of B cell activation (Figures 4A,B). Meanwhile, genes negatively correlated with cytolytic T-cell activity were related to normal biology activities, such as chemical synaptic transmission and regulation of ion transmembrane transport, etc. These results indicated that samples in CYT-high group always possessed a more complex and strongly immune response system.

To our knowledge, immune checkpoints were crucial for suppress unwanted and harmful self-directed activities and modulating the duration of immune system. However, in cancer, immune checkpoints could shut down the T-cell powerful fighting activities, allowing tumor to grow undisturbed. Then we explore the relationship of CYT and checkpoints expression (CTLA4, IDO1, LAG3, PD1, PD-L1, PD-L2, TIGIT, and TIM-3). The findings were intriguing, both in CGGA and TCGA datasets, all checkpoints were significantly positively associated with T-cell cytolytic activity (Figures 4C,D). These results indicated that checkpoints were induced more as immune suppressors in CYT-high group in tumor microenvironment when immune cell and immune response were more active. These data provided significantly evidence that CYT may be the representation of immune checkpoints synergistically effect in glioma and might play an important role in the immunosuppressive response through a series of immune checkpoints.

### CYT Index Was Positively Related to Peri-Tumor Edema

**Figure 5:**
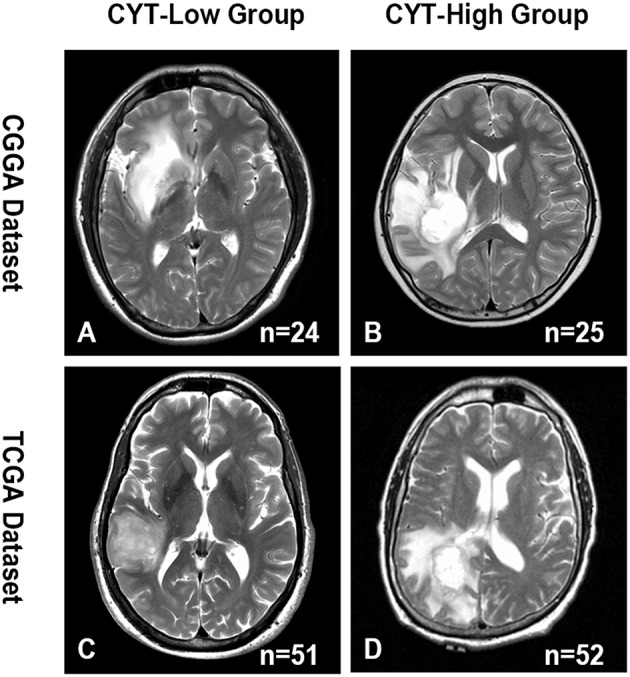
Representative images of the difference in the extent of peritumoral edema between CYT-low group and CYT-high group. CYT-high group significantly possessed more-severe edema than patients in CYT-low group in CGGA **(A,B)** and TCGA **(C,D)** dataset.

Edema, a reflection of inflammation, was a common pathophysiological entity surrounding gliomas. The secretory products of macrophages were reported might contribute to the formation of glioma edema. Then, we compared the edema extending differences between CYT-low and CYT-high group to investigate the potential association of CYT and peritumoral edema. As shown in Figure 5, we demonstrated that gliomas in CYT-high group significantly possessed more-severe edema than other patients with lower cytolytic T-cell activity in CGGA (*p* = 0.0003) and TCGA dataset (*p* = 0.0002), respectively. And we validated this intriguing phenomenon in an independent validation group (*p* = 0.04). Those results suggested peritumoral brain edema probably was a reflection of CYT and tumor immune response.

### CYT Predicted Unfavorable Prognosis

**Figure 6:**
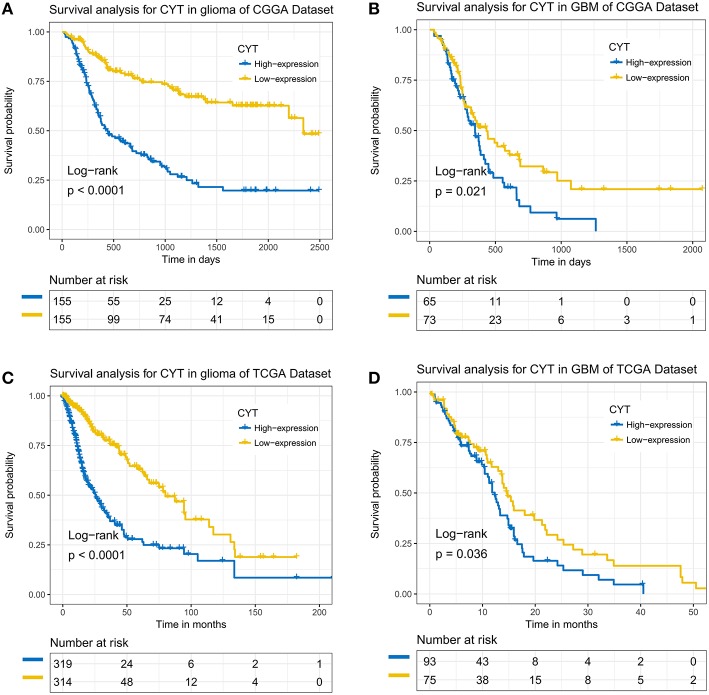
The immune cytolytic activity survival curves of glioma and glioblastoma. Kaplan–Meier survival analysis showed that a higher level of CYT conferred a significantly worse prognosis in glioma and glioblastoma patients in CGGA **(A,B)** and TCGA **(C,D)** cohorts, respectively.

To further analysis the prognostic value of CYT, we employed dichotomization to separate cases for depicting the Kaplan Meier survival curves according to the cutoff value (median CYT value). In CGGA dataset, we found that patients with higher CYT value had a significant shorter overall survival than their counterparts in glioma (*p* < 0.0001) and GBM (*p* = 0.021) (Figures 6A,B). Simulation results were shown in TCGA dataset that CYT value was a dismal biomarker in glioma (*p* < 0.0001) and GBM (*p* = 0.036) (Figures 6C,D). Next, we conducted cox regression model to evaluate the prognostic value of CYT and other prognostic factors. In CGGA dataset (Figure S4A), the result confirmed that CYT value was significantly associated with overall survival (*p* < 0.0001) after adjusting other factors, such as age at diagnosis (*p* = 0.54), grade (*p* < 0.001), IDH status (*p* = 0.09), and 1p19q status (*p* < 0.001). In TCGA dataset (Figure S4B), the result suggested that CYT value (*p* = 0.04) was an independent prognostic indicator. These results demonstrated that CYT value was an independent unfavorable predictor for patients with glioma.

## Discussion

The immune system played pivotal roles in preventing and combating tumor (18). Recently, cancer immunotherapy which recover the ability of immune system to recognize and eliminate the tumor cell have shown striking clinical results, but only for a few kinds of tumors (8, 19). The treatment response heterogeneity of patients may reflect the inter-tumor and the inter-individual variability. PD-1 and CTLA4 were two well-known checkpoint inhibitor which were expressed on T cell surface blocking checkpoint receptors to suppress T cell activation (20, 21). The effectiveness that clinical trials of anti-PD-1 and anti-CTLA-4 monotherapy for glioma patients were limited (22). Therefore, new treatment concepts and therapeutic approaches are urgently needed (23). In this study, we enrolled 1,024 glioma samples from CGGA and TCGA datasets and calculated the CYT values which could reflect CD8+ T cell activity in following analysis (10). We found that CYT values were significantly upregulated in higher grade gliomas and IDH wild-type gliomas, indicating that CYT was associated with more malignance glioma biological progress. Additionally, CYT was upregulated in mesenchymal subtype and could predicted the mesenchymal subtype of glioma with dramatic accuracy. Those results indicated that GBM patients exhibit a higher immune-activity than LGG which were controversially with previous studies (10, 24). Moreover, the association of CYT and IDH wild-type gliomas may lead to a statement of immune-activation, therefore altering the glioma microenvironment (25).

Then we performed an integrated analysis the mutation profile of glioma in the context of CYT value. We found that mutations of IDH, ATRX, TP53, CIC, FUBP1, and 1p/19q co-deletion were more common in CYT-low group. IDH mutations was considered as the truncal event and occurred in the vast majority lower grade glioma and secondary GBM. ATRX and TP53 were reported as the driver mutations for astrocytoma (26, 27), whereas the driver events for oligodendroglioma were 1p/19q co-deletion, CIC, and FUBP1 mutations (28). The genes significantly mutant in glioma of CYT-high group were mainly involved in RTK-RAS-PI3K and ERK pathways. RTK-RAS-PI3K and ERK pathways were correlated with cellar functions, such as cell growth, proliferation, differentiation, motility, survival and intracellular trafficking (16). Upregulated RTK-RAS-PI3K and ERK pathways will promote tumor progression and chemoresistance (29). The mutation of KEL (30), TTN (31), and SPTA1 (32) resulted in a variety of hereditary red blood cell and neutrophils disorder which could induced an abnormal immune response in human body. Mutations in SWI/SNF and chromatin-remodeling complex (SMARCA4, COL6A3, RYR2, and SPEG) contributed to cancer and neurological disorders (33). CHD9 (34) and DNAH3 (35) encoded large proteins that are constituents of the microtubule-associated motor protein complex. The mutations will affect components or regulators of the nexin–dynein regulatory complexes which could cause microtubule disarrangements and ATPase activity abnormally. Moreover, HMCN1 encoded a large extracellular member of the immunoglobulin superfamily (36). MUC17 played an inhibitory role in cell-cell and cell-stroma interactions and in cytotoxic immunity (37). The mutation of HMCN1 and MUC17 may induce the increased cytolytic activity of glioma. Beyond strong associations between gene mutations and cytolytic activity, we also revealed that higher chromosome aberrations were notably prominent in CYT-high group gliomas. Beyond strong associations between cytolytic index and genomic structural variation, our analysis revealed an underappreciated link between genomic alteration and immune activation in glioma, suggesting that the genomic structural variations may be the fundamentally events leading the difference of immune response.

The gene ontology analysis showed a positive correlation of CYT and immune response, regulation of cell angiogenesis and adhesion, and immune cells activation. Meanwhile, we found that the expression level of several immune-checkpoint molecules, including CTLA-4, IDO1, LAG3, CD274, PD-1, PD-L2, TIGIT, and HAVCR2 were significantly positively associated with cytolytic activity in glioma. CYT could be a useful biomarker for glioma patients to predict the responsiveness to immune checkpoint blockade therapy (10). Such immune checkpoints possibly played a synergistic effect in cancer immunotherapy, we expected that CYT might also be a predictor for combinatorial target therapy.

Peri-tumoral edema, a common symptom caused by inflammation response, caused mass effect and was associated with poor prognosis in glioma patients. We found that patients in CYT-high cohort were significantly associated with greater extend of edema than in CYT-low cohort. Our result indicated that the MRI image may potentially provide a simple approach to reflect the host immune status and the effective of immunotherapy.

Previously pan-cancer research shown that higher expression of CYT value was correlated with improved prognosis. However, we found that CYT value was an independent unfavorable prognostic factor for glioma patients. This prognostic value was also observed when taking GBM as a separated type of glioma. This controversial result may be the reflection of tumor heterogeneity (10). The positive correlation between CYT and immune functions indicated that an enhanced local host immune response contributed to unfavorable prognosis, consistent with previously report that the local immune phenotype in GBM was the most dominated by the responses of invasion, angiogenesis, and proliferation (38).

In summary, we explored the clinical roles and immune biological processes of CYT in more than 1,000 diffuse gliomas. Calculating CYT may help us elucidate the responses driven by the immune microenvironment more completely and extend our understanding of immunotherapy strategies in glioma.

## Author Contributions

ZLW made substantial contributions to conception, design, and wrote the manuscript. ZW, GZL, ZSB, and QWW gave suggestions on study design, discussed, and interpreted the data. TJ and CBZ designed and supervised study. All authors read and approved the final manuscript.

### Conflict of Interest Statement

The authors declare that the research was conducted in the absence of any commercial or financial relationships that could be construed as a potential conflict of interest.

## Supplementary Material

Figure S1 The distribution of GZMA, PRF1, and CYT within glioma in CGGA and TCGA datasets.

Figure S2 Immune cytolytic activity was significantly increased in gliomas with IDH wild type and 1p/19q intact groups in CGGA **(A,C)** and TCGA **(B,D)**.

Figure S3 The different genes between CYT-low and CYT-high groups in CGGA **(A)** and TCGA **(B)** dataset. Significantly upregulated genes are colored in red, whereas significantly downregulated genes are colored in blue.

Figure S4 Forest plot of hazard ratios for overall survival assessed by CYT level and clinicopathological factors. CYT was an independent prognostic factor in CGGA **(A)** and TCGA **(B)** datasets.

Table S1 The core genes for the mesenchymal subtype glioma.
