# Supplementary figures and images for: Immune Cytolytic Activity Is Associated With Genetic and Clinical Properties of Glioma
Source: Front Immunol. 2019 Aug 2;10:1756. doi: 10.3389/fimmu.2019.01756 (PMC6688525; doi:10.3389/fimmu.2019.01756)

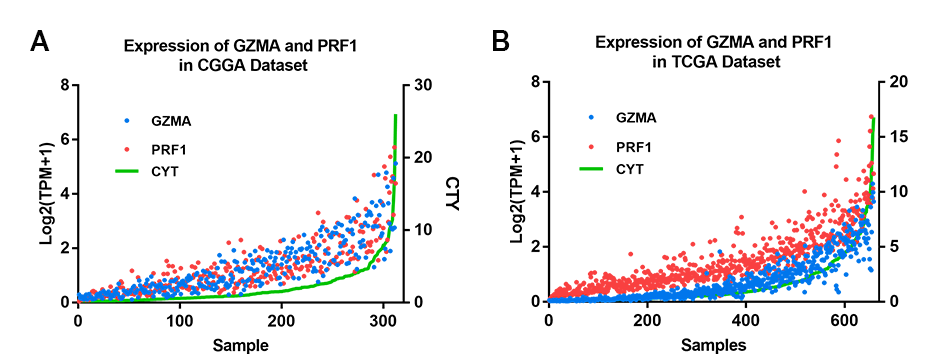

Supplement: Figure S1 — The distribution of GZMA, PRF1, and CYT within glioma in CGGA and TCGA datasets. [file Image_1.TIF]

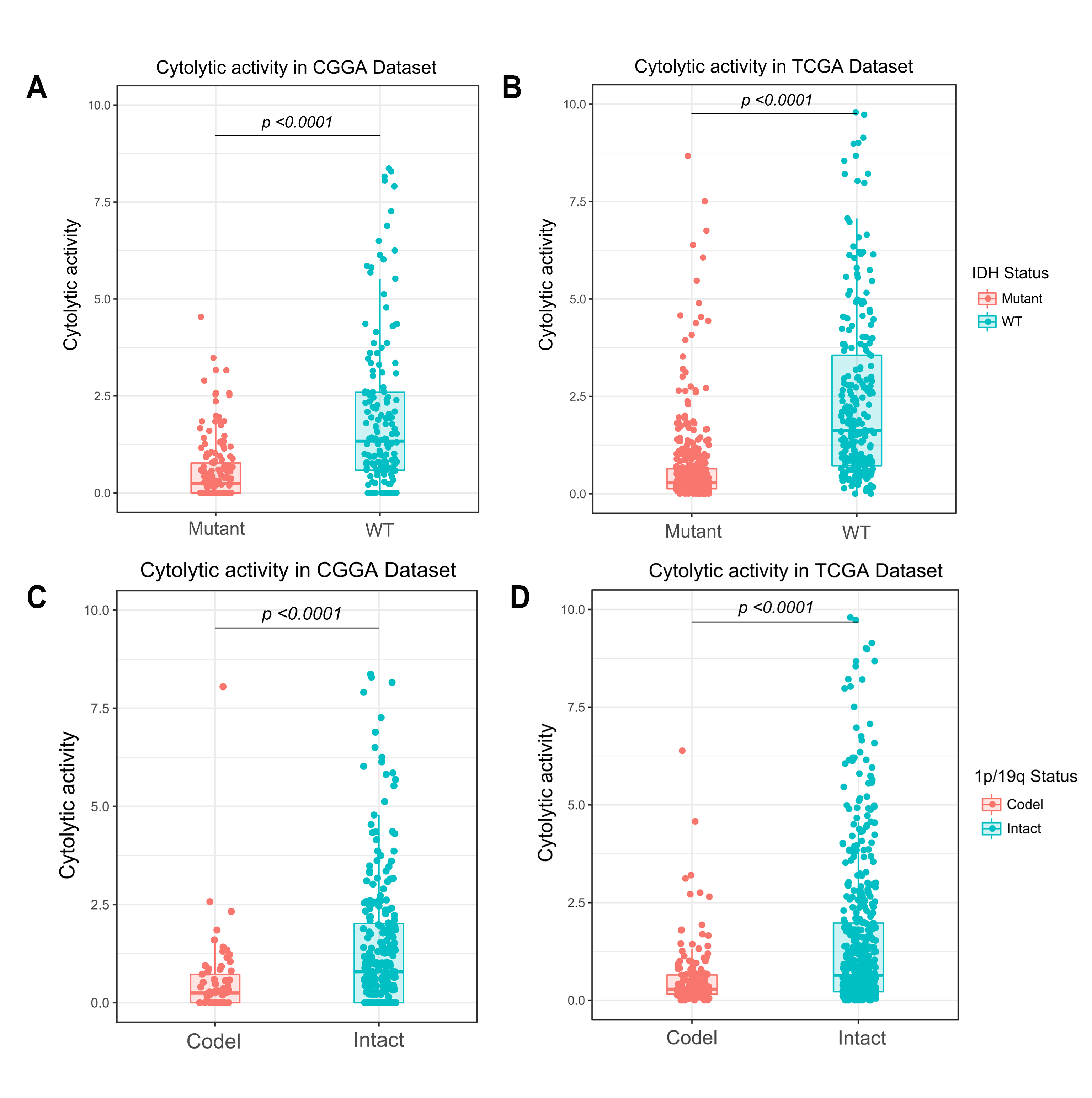

Supplement: Figure S2 — Immune cytolytic activity was significantly increased in gliomas with IDH wild type and 1p/19q intact groups in CGGA (A,C) and TCGA (B,D). [file Image_2.TIF]

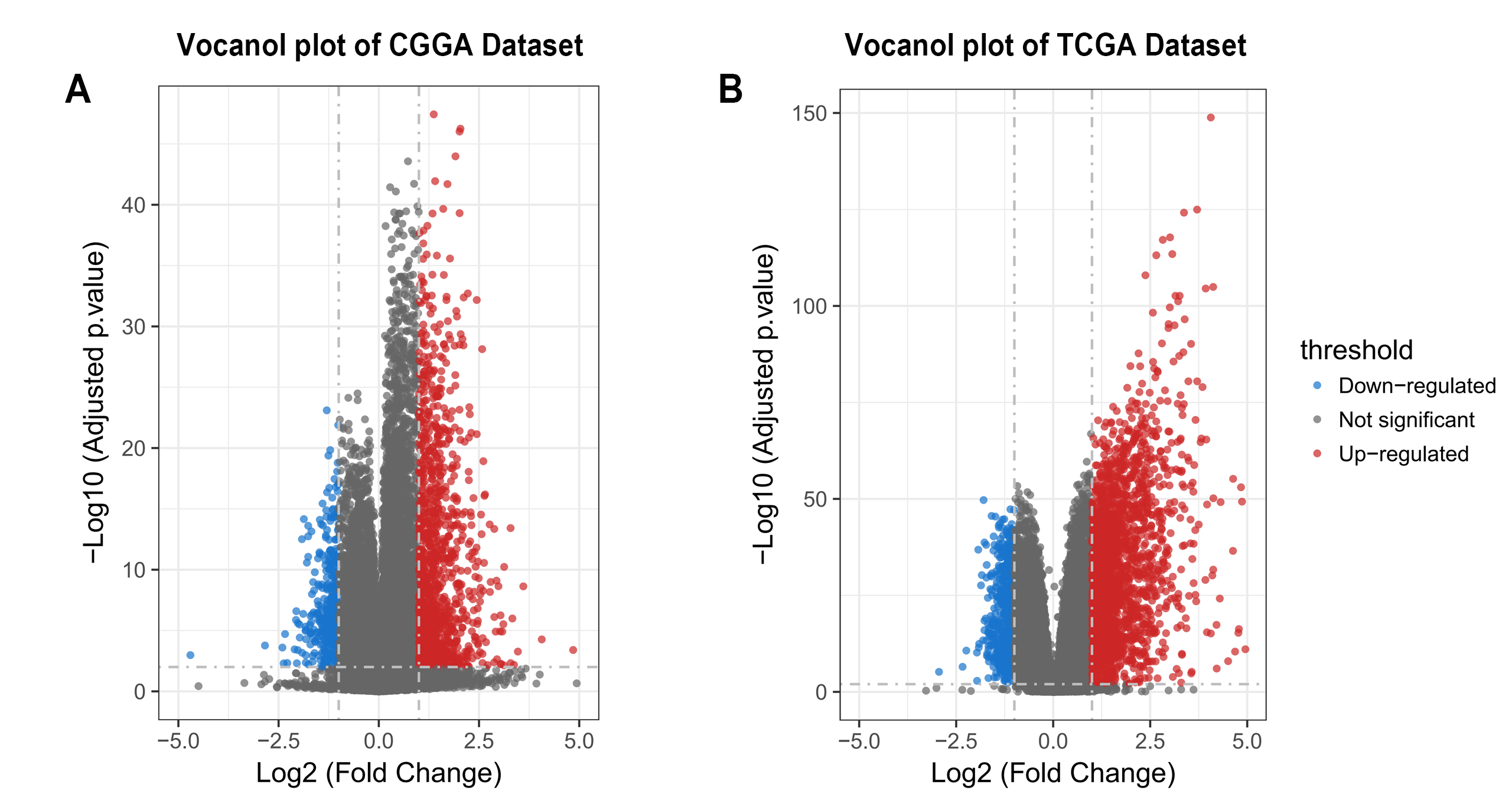

Supplement: Figure S3 — The different genes between CYT-low and CYT-high groups in CGGA (A) and TCGA (B) dataset. Significantly upregulated genes are colored in red, whereas significantly downregulated genes are colored in blue. [file Image_3.TIF]

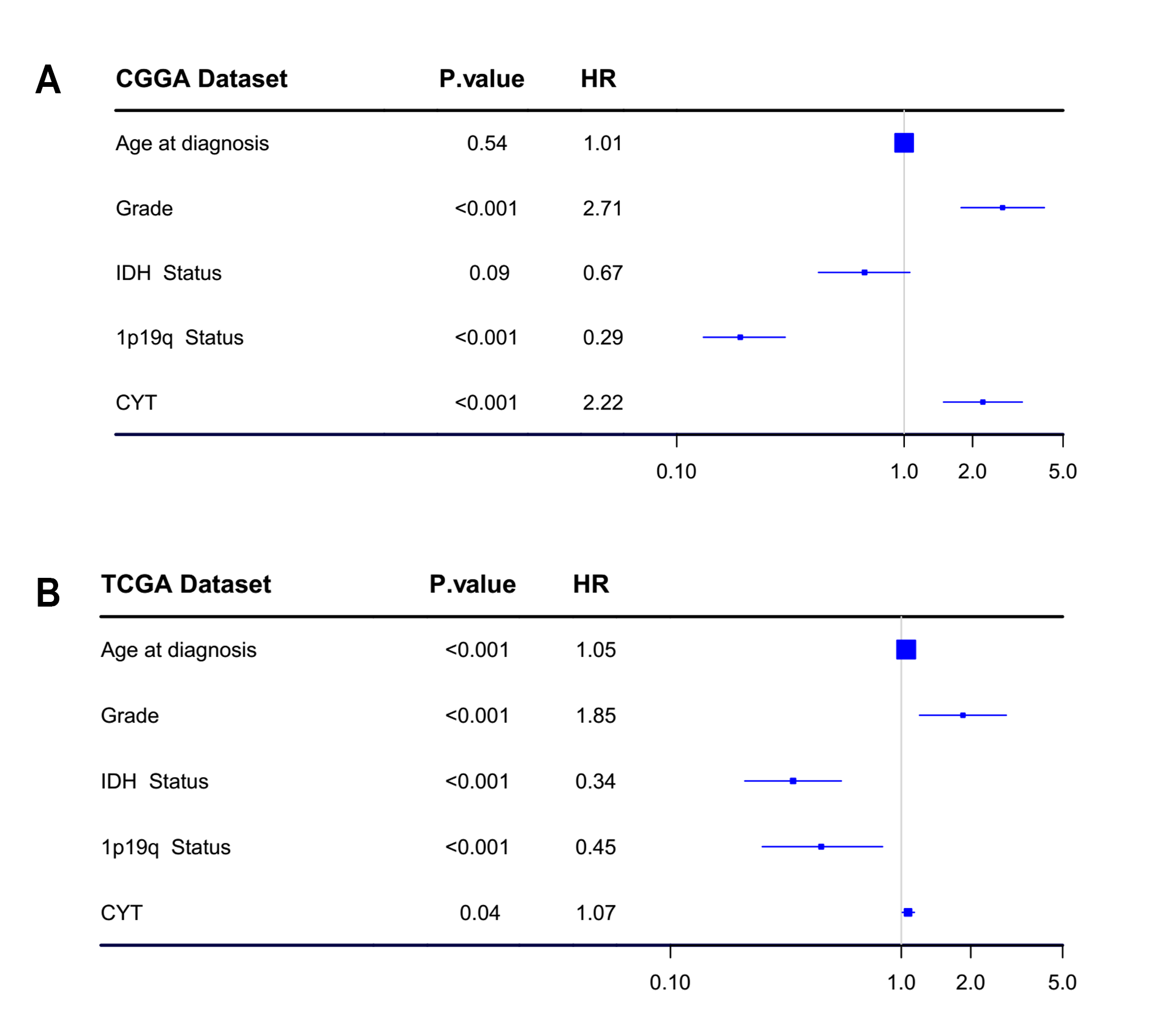

Supplement: Figure S4 — Forest plot of hazard ratios for overall survival assessed by CYT level and clinicopathological factors. CYT was an independent prognostic factor in CGGA (A) and TCGA (B) datasets. [file Image_4.TIF]
